# Improving Sensitivity in Raman Imaging for Thin Layered and Powdered Food Analysis Utilizing a Reflection Mirror

**DOI:** 10.3390/s19122698

**Published:** 2019-06-15

**Authors:** Santosh Lohumi, Moon S. Kim, Jianwei Qin, Byoung-Kwan Cho

**Affiliations:** 1 Department of Biosystems Machinery Engineering, College of Agricultural and Life Science, Chungnam National University, 99 Daehak-ro, Yuseong-gu, Daejeon 34134, Korea; santosh123@cnu.ac.kr; 2 Environmental Microbial and Food Safety Laboratory, Agricultural Research Service, U.S. Department of Agriculture, Powder Mill Rd. Bldg. 303 BARC East, Beltsville, MD 20705, USA; Moon.kim@ars.usda.gov (M.S.K.); Jianwei.qin@ars.usda.gov (J.Q.)

**Keywords:** Raman imaging, reflection mirror, layered samples, subsurface analysis, food authenticity

## Abstract

Raman imaging has been proven to be a powerful analytical technique for the characterization and visualization of chemical components in a range of products, particularly in the food and pharmaceutical industries. The conventional backscattering Raman imaging technique for the spatial analysis of a deep layer suffers from the presence of intense fluorescent and Raman signals originating from the surface layer which mask the weaker subsurface signals. Here, we demonstrated the application of a new reflection amplifying method using a background mirror as a sample holder to increase the Raman signals from a deep layer. The approach is conceptually demonstrated on enhancing the Raman signals from the subsurface layer. Results show that when bilayer samples are scanned on a reflection mirror, the average signals increase 1.62 times for the intense band at 476 cm^−1^ of starch powder, and average increases of 2.04 times (for the band at 672 cm^−1^) for a subsurface layer of high Raman sensitive melamine powder under a 1 mm thick teflon sheet. The method was then applied successfully to detect noninvasively the presence of small polystyrene pieces buried under a 2 mm thick layer of food powder (a case of powdered food adulteration) which otherwise are inaccessible to conventional backscattering Raman imaging. In addition, the increase in the Raman signal to noise ratio when measuring samples on a mirror is an important feature in many applications where high-throughput imaging is of interest. This concept is also applicable in an analogous manner to other disciplines, such as pharmaceutical where the Raman signals from deeper zones are typically, substantially diluted due to the interference from the surface layer.

## 1. Introduction

Raman spectroscopy with an imaging component called Raman imaging has become a versatile analytical technique with wide use, spanning from agro-food and pharmaceutical analysis, biological and biochemical sectors, forensic science, material science analysis, and art work, to name a few [1,2,3,4]. The main advantage of Raman spectroscopy over near-infrared (NIR) spectroscopy is that Raman spectra show distinct and well-resolved peaks, and a big advantage of using Raman spectroscopy over infrared (IR) is that the sample preparation is much easier and less time consuming. Despite its advantage, the major drawback of Raman spectroscopy is the fact that the Raman scattering is usually weak, thus it suffers from low signal intensity. In general, Raman signal intensity can be conventionally improved by using a high power laser source and/or by increasing the exposure time, thus allowing more photons to reach the detector. However, these typical solutions can lead to sample destruction and prolong the scanning process, respectively. Resonance Raman spectroscopy is another way to increase the Raman signals by choosing an excitation (laser) wavelength carefully to match with the electronic absorption bands of a compound or material of interest. Such a combination can result in scattering intensities which increase several fold [5]. Since this technique is sample-centric, it cannot be considered a global solution of the aforementioned problems associated with Raman spectroscopy.

In Raman spectroscopy, when a monochromatic laser beam hits the sample, the scatter Raman signals as well as the Rayleigh photons occur over all directions, and thus measurements can be made from any position according to the user requirements. The traditional and most convenient way of doing Raman analysis is by back scattering geometry, where the incident light is focused on the sample and the backscattered radiation is collected by the same objective. In the conventional back scattering geometry of Raman spectroscopy, the collected Raman signals are highly representative of the surface constituent, and thus, there is a greater spectral contribution from the surface of the sample. Raman spectroscopy and imaging in backscattering geometry has become one of the most widespread tools for chemically specific analysis and characterization of food, pharmaceutical, and biological materials. However, these materials can be made up of layers of different chemical constituents. In particular, adulterants in food samples can settle down to the bottom of the sample, or pharmaceutical tablet samples made up of different chemical constituents and polished with a superficial layer of coating material. Therefore, with the use of backscattering geometry, the superficial layer of the sample often overwhelms the weaker Raman signals of the bottom material.

Transmittance Raman spectroscopy can be used to preferentially detect photons originating from a deeper layer of a turbid sample. Therefore, transmittance Raman has been utilized in the pharmaceutical sector for the analysis of whole tablets and capsules with applications to the quantitative assessment of active pharmaceutical ingredients (APIs) [6,7,8]. However, visualization of the chemical constituents of a sample is equally important to confirm product uniformity. Despite the above advantage of transmittance Raman, the instrumental complexity required to acquire the chemical images of powder and turbid samples remain an obstacle to the widespread application. Hence, owing to the instrumental simplicity and ease of use, backscattering Raman is still preferred for powdered food and pharmaceutical samples and semiliquid biological samples when chemical images are of interest.

**Figure 1:**
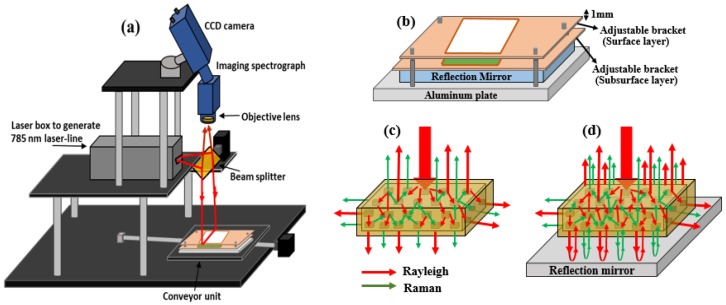
Schematic diagram of line-scan Raman imaging system (**a**), sample holder with reflection mirror to facilitate layered samples (**b**), and schematic representation of Raman signals collated without (**c**) and with a back-reflection mirror (**d**).

In general, conventional backscattering Raman spectroscopy suffers from low photon fluxes, and the achievement of sufficient signal-to-noise (S/N) ratios at acceptable acquisition times often require the use of high-throughput detection systems and relatively high laser power. This is the case in weakly scattering Raman media, in deep laser Raman spectroscopy or in the presence of an intense fluorescent background, which can severely reduce Raman S/N ratios [9]. The S/N ratio of Raman signals can be improved by increasing the number of photons reaching the detector. As was mentioned above, the Raman light scatter direction is random, therefore, by placing a reflective object under the sample while using backscattering geometry, the Raman photons transmitted through the sample can be reflected back toward the detector, as is in the case of transflectance spectroscopy (Figure 1). Thus, the higher number of photons reaching the detector will ultimately contribute to Raman signals and as a result, Raman signals can be enhanced. It should also be noted that the element will naturally lead not only to an enhancement of Raman signals but also to an increase of any fluorescent background in the same portion. However, in comparison to the excitation (Rayleigh) photons, Raman scatter photons are more often generated inside, and propagate and exit the sample over a relatively wide distribution area around the sample because of the many successive scattering events [10]. Here, we used the principle of transflectance spectroscopy to reflect the transmitted photons back to the detector by means of a reflection mirror placed under the sample, thus enhancing the Raman signals and facilitating the visualization of subsurface materials buried underneath the superficial layer. However, until now, this approach was applied only to biological liquid or semiliquid samples to improve the Raman signals of tissues and cells using a mirrored stainless steel substrate [11,12]. 

Most recently, Raman imaging on both the micro- and macroscale levels have been successfully tested for powdered food authenticity analysis [13,14,15,16,17,18,19]. Since a laser beam can penetrate a certain depth into a sample, the penetration depth was first determined to ensure that the adulterant particles at the very bottom of the sample could be detected [18,20]. In practice, a thick layer of adulterant will obviously generate a higher number of Raman photons than in the case of food adulteration. In this case, a reflection mirror can be utilized to enhance the Raman signals from the adulterant particles settled at the bottom, to effectively detect the authenticity of powdered food materials with high instrumental sensitivity.

In this study, we demonstrate the ability to retrieve information from the subsurface layers which would otherwise be inaccessible to conventional back scattering Raman imaging. We first provide experimental results on solid powders covered with a thin sheet of Teflon. The second specimen was used to mimic the real situation with food authenticity analysis. We have also observed that when the Raman images of layered samples were collected utilizing a reflection mirror, a significant enhancement in Raman signals from the bottom layer was observed, however, no notable enhancement from the superficial layer was noticed.

## 2. Materials and Methods

### 2.1. Reflection Mirror

Silver-coated reflection mirrors which offer high reflectance in the visible and NIR ranges were purchased from ThorLabs (https://www.thorlabs.com). The reflection mirror (PFSQ20-03-P01, ThorLabs, Hans-Boeckler, Dachau, Germany) was 50.8 mm × 50.8 mm × 6.0 mm in size with a clear aperture >90% of the dimension. To protect the silver-coated mirror from oxidation, it has a durable SiO_2_ overcoat with an approximate thickness of 100 nm. According to the manufacturer, these silver-coated mirrors offer the highest reflectance in the visible-NIR spectrum of any metallic mirror, and the measured average reflectance is >97.5% for 450–2000 nm (https://www.thorlabs.com). This wave range is compatible with our 785 nm-based line-scan Raman imaging system, as the system covers a spectral range 740.6 to 1010 nm.

### 2.2. Specimens

As a demonstration of the effectiveness of the reflection mirror to increase the sensitivity of Raman imaging, we first prepared the layered samples. Specimen-1 (S1) consisted of two layers, a subsurface layer made up of 1 mm thick high background fluorescence starch powder covered by a 1 mm thick Teflon sheet (50 mm × 50 mm). The S2 subsurface layer was made up of highly Raman sensitive and low fluorescence background melamine powder overlapped with a 1 mm thick Teflon sheet. A Teflon sheet was selected as the surface material to provide a uniform superficial layer without disturbing the uniformity and thickness of the subsurface layer of powder. The teflon and food powder samples used in this study to demonstrate the application of mirror-based methodology were nearly opaque.

In S3, five polystyrene crystals (approximately 3 mm × 3 mm × 2 mm in size) were placed in five difference locations under the 2 mm thick surface layer of wheat flour. 

S4 was prepared to mimic the real-world powdered food adulteration situations where small particles of hazardous materials are often added to food powder. For this purpose, small and irregular-shaped polystyrene particles of different sizes and thicknesses were placed under a 2 mm thick layer of wheat flour. The smallest and thinnest (~140 µm) particle was at the center, and four particles were placed along the four edges, where two thick (~230 µm) particles were at the upper left and lower right sides of the sample.

Specimens S1 and S2 were prepared to evaluate the effect of the back reflection mirror in layered samples. S3 was prepared to confirm the penetration depth of the laser line in wheat flour. S4 aims to show the effectiveness of the reflection mirror as a sample holder to increase the sensitivity of Raman imaging by back-reflecting the Raman photons from the small particles buried under a thick layer of high fluorescence background food powder, which is the case with powdered food adulteration.

All specimens excluding S3 were measured both with and without the reflection mirror, and two replicates for each sample in each case were collected. The sample holder used was an aluminum plate with attachable brackets of different thicknesses and sizes to achieve the desired thickness of layered samples (Figure 1). In the other case, the reflection mirror was placed on the aluminum plate and the specimen (S1, S2, or S3) was placed over the reflection mirror with the help of the attachable brackets. Special care was taken while preparing S4, as the pieces of polystyrene were first placed onto the sample holder and wheat flour was then poured on the polystyrene pieces using a 250 µm sieve, and the powder surface was leveled without pressure at the top edge of the sample holder using a spatula.

### 2.3. Raman Imaging Instrumentation and Measurements

Raman imaging measurements were carried out on the 785 nm-based line-scan Raman imaging system illustrated in Figure 1a. A 785 nm laser line was used as the illumination source and the uniformity of the laser line was insured by incorporating an engineered diffuser (ED1-L4100; ThorLabs, Hans-Boeckler, Dachau, Germany). A 785 nm diachronic beam splitter (Semrock, Rochester, NY, USA) was mounted on an xyz translation stage incident at 45° to reflect the laser line for normal incidence on the sample surface. The generated Raman signals passed through the beam splitter and were collected by the sensing module consisting of an objective lens of 23 mm focal length (Schneider Optics, Xenoplan 1.4/23 mm NY, USA). For aperture and focus adjustment, two 785 nm longpass filters were used to mitigate the Rayleigh effect, and an imaging spectrograph was used to disperse the incoming signals onto the CCD detector. The CCD camera (iKon-M 934, Andor Technology, South Windsor, CN, USA) was thermoelectrically cooled to −65°C to minimize the dark current effect during image acquisition. The camera was connected to a computer via USB cables for control and data transfer. A computer-controlled stepper motor-based translation stage (Velmex, Model XN 10- 0180-M02-21, NY, USA) was integrated to move the samples during line-scanning. A detailed description of the system development can be found in our previous studies [13,21].

The developed line-scan Raman imaging system covers a spectral range of 740–1010 nm corresponding to Raman shifts of −763–2837 cm^−1^. The sample surface to beam splitter distance was set to 16 cm. Thus, the generated laser line on the sample surface was approximately 1.5 mm thick and 140 mm wide, and the power of the laser line on the sample surface was approximately 12 mW/mm^2^ as measured by an S314C thermal power sensor connected to digital power meter (PM 100D, ThorLabs, Hans-Boeckler, Dachau, Germany). The camera lens to sample distance was set to 24 cm, thus the instantaneous field of view (IFOV) of the Raman system was determined to be 148 mm and provided a nominal pixel size of approximately 0.145 mm along the IFOV. The CCD exposure time was set to 2 s for S1 considering the low Raman signals from subsurface layer of starch, and 1 s for all other specimens, and a step size of 0.15 mm/scan was selected to cover the spatial shape of the samples. Since it is obvious that the laser line power on the sample surface increases as the distance between the beam-splitter and sample decreases, the result is a slightly higher penetration depth of the laser. Therefore, a height adjustable stage was used to compensate for the thickness of the mirror (6 mm), thus maintaining the same distance while collecting data with or without the mirror.

Raman maps of all specimens (S1 to S4) were acquired with the same instrumental setting and the Raman data was saved in 3D format. In addition to the aforementioned specimens, Raman data of starch, melamine, Teflon, and polystyrene were also collected for reference. In addition, dark current images were collected with the laser off and cap covering the lens, and subtracted from the original sample data. Only the corrected Raman data was used for further analysis.

### 2.4. Data Processing and Analysis

Raman data was analyzed using MATLAB software (MathWorks, Natick, MA, USA). A spatial region of interest (ROI) was selected for each sample and the data size was further reduced in spectral dimension by keeping only the informative spectral range from 360 and 1800 cm^−1^. The selected Raman data were then reshaped in 2D format and the fluorescence background was corrected using a baseline correction method based on the adaptive iteratively reweighted penalized least square (airPLS) method [22]. The effect of cosmic rays (sharp spikes) and high-frequency noise in the fluorescence-corrected Raman data were removed using a median filter with a 3 × 3 moving window, which ultimately smoothed the dataset.

The Raman spectra of each reference material was extracted from the preprocessed data set and a univariate method, which utilizes a single band image intensity from the 3D hypercube, was adopted and image intensity bar was set to visualize the spectral intensity and spatial position of the aforementioned materials positioned in the surface or subsurface layer of each scanned specimen. Specimen S3 was analyzed using the spectral angle mapper (SAM) method [23]. SAM analysis is a supervised method which utilizes the reference spectrum of the endmember (polystyrene) to calculate the angle between the pixels of the endmember and target material. The SAM generated rule images were used to visualize the spatial location of hidden polystyrene crystals under a 2 mm thick superficial layer of wheat flour. For S4, the Raman chemical images of polystyrene particles under wheat flour were generated using both the unsupervised and supervised analysis method. Principle component analysis (PCA) was first executed to Raman data of S4 collected with and without the reflection mirror, and the SAM method as discussed above was applied as the supervised analysis method. The generated Raman chemical images of the polystyrene particles for data collected with the reflection mirror were then compared with data collected without the mirror, and thus the effectiveness of the reflection mirror as a sample holder was demonstrated.

## 3. Results and Discussion

The first conceptual demonstration of the effectiveness of the reflection mirror to improve the Raman signals was performed on two different two-layer samples (S1 & S2), composed of a 1 mm thick layer of starch (S1) or melamine (S2) covered with a 1 mm thick Teflon slice. As it has been stated that the conventional backscattering Raman spectroscopy is only suitable for surface or minimal subsurface analysis [24], the greater contribution to the Raman signals is from the superficial layer. If the material in the bottom layer of the sample is less Raman sensitive, then weak Raman signals originating from the bottom layer will possibly be overwhelmed by either the fluorescence or noise generated from the superficial layer. Therefore, the main complication of how to enhance the Raman signal of the bottom layer in such a way to ensure a decent signal-to noise (S/N) ratio suitable for qualitative or quantitative evaluation of the subsurface layer by chemometric analysis should be addressed. Thus, as a possible solution to aforementioned limitation of Raman spectroscopy, we utilize a reflection mirror to reflect the transmitting Raman signals back to the detector. Teflon was used as a superficial layer because the Raman peaks of Teflon do not interfere with the Raman signals of the chosen subsurface materials.

**Figure 2:**
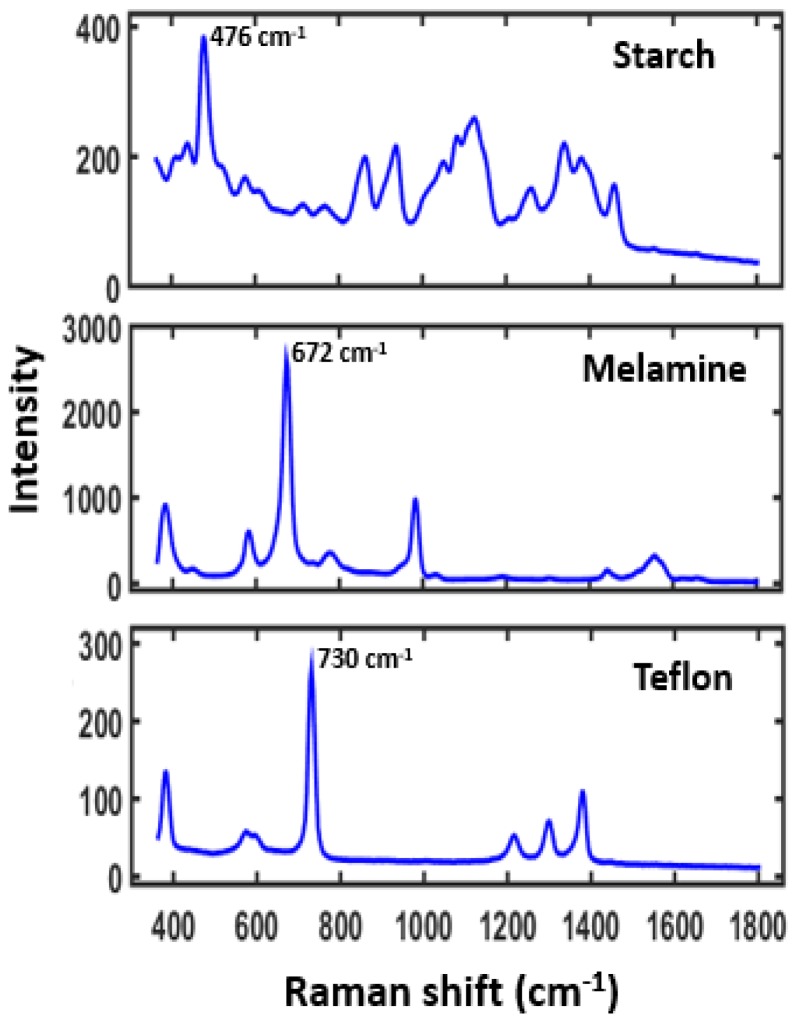
Raman spectra of starch, melamine, and Teflon used in this study to form subsurface and surface layers.

The reference Raman spectra of surface and subsurface materials used in this study (S1 & S2) are shown in Figure 2. The Raman spectrum of starch shows high background fluorescence and low Raman intensity peaks. However, the Raman spectrum of melamine shows relatively no background fluorescence and high Raman peaks. The surface layer Teflon shows a decent Raman peak at 730 cm^−1^, free from any interference peaks from the subsurface layer, and thus this band was further utilized for the generation of single band images of the surface layer.

**Figure 3:**
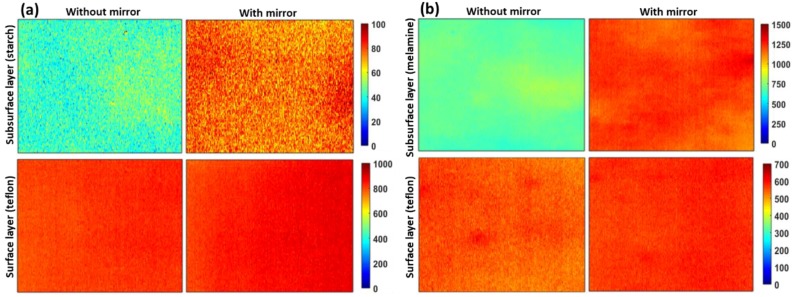
Preprocessed Raman band images of S1 (**a**), and S2 (**b**) for replication 1. Raman signals increase for the subsurface layer with the mirror (difference between the images on upper row), however, there was no notable difference in the Raman intensity of the surface layer when using the reflection mirror (bottom row). Raman maps with and without mirror are presented in the same intensity scale for the purpose of direct comparison and the intensity scales are in arbitrary units.

**Figure 4:**
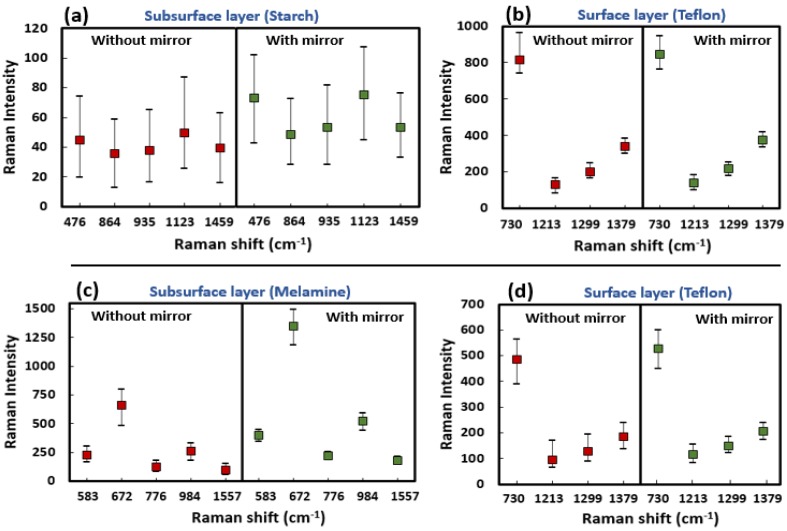
Box plots showing the distribution of magnitudes of Raman signal increases for the subsurface layer on a mirror: subsurface (**a**) and surface layer (**b**) of S1, and subsurface (**c**) and surface layer (**d**) of S2. (This figure is the box plot representation of Figure 3 with additional wavebands).

The preprocessed Raman band images (as marked in Figure 2) of both surface and subsurface materials for both S1 & S2 are shown in Figure 3a,b, respectively. The conventional Raman imaging data acquired without the reflection mirror shows comparatively lower intensity for the subsurface layer than those data collected with the reflection mirror. However, it is worth mentioning there are no notable differences in Raman intensity with respect to the surface layer of Teflon. This is also apparent from the plot (Figure 4) for the comparison of Raman intensities of the specific peaks of surface and subsurface layers of S1 and S2 samples scanned with and without the reflection mirror. To easily convey the increase in Raman signals from the subsurface layer, a Raman improvement factor was calculated as the average of the ratio of the peak intensity for each identical Raman peak of preprocessed Raman data collected with and without the reflection mirror.

For S1, it was observed that the average Raman signal intensity of the bottom layer increased by an improvement factor of between 1.35 and 1.62 for 1459 cm^−1^ and 476 cm^−1^, respectively, among five selected peaks of starch (Figure 4a). In contrast, when the Raman images were acquired with the mirror, no notable improvement in Raman intensity of the superficial layer was observed.

For the next sample (S2), Raman imaging data of melamine powder under Teflon were analyzed in same manner as for S1. In S2, melamine was used as a base material owing to its high Raman sensitivity and fluorescence free background (unlike starch), to test the effectiveness of using a reflection mirror in the case of a highly Raman sensitive bottom layer. For the most intense Raman peak of melamine at 672 cm^−1^, a maximum Raman improvement factor of 2.04 was calculated, with a minimum improvement factor of 1.75 at 583 cm^−1^. However, unlike the case of S1, a small improvement in peak intensity of the superficial layer was observed (Figure 3b and Figure 4d). This is probably because the peak in the Raman spectrum of melamine in the region of 672 cm^−1^ and a small bump approximately 780 cm^−1^ shifted slightly upward when the reflection mirror was used, thus resulting in a small increase in the Teflon peak at 730 cm^−1^. In addition, similar results were observed for second replication where Raman peak of starch (476 cm^−1^) and melamine (672 cm^−1^) showing a maximum improvement factor of 1.6 and 1.99, respectively, when a mirror was used. We would like to note that the Raman intensity of the surface layer of Teflon for specimen S1 is higher than for S2 (in Figure 3). This is because of the S1 data collected with an exposure time of 2 s, whereas S2 data collected with an exposure time of 1 s.

**Figure 5:**
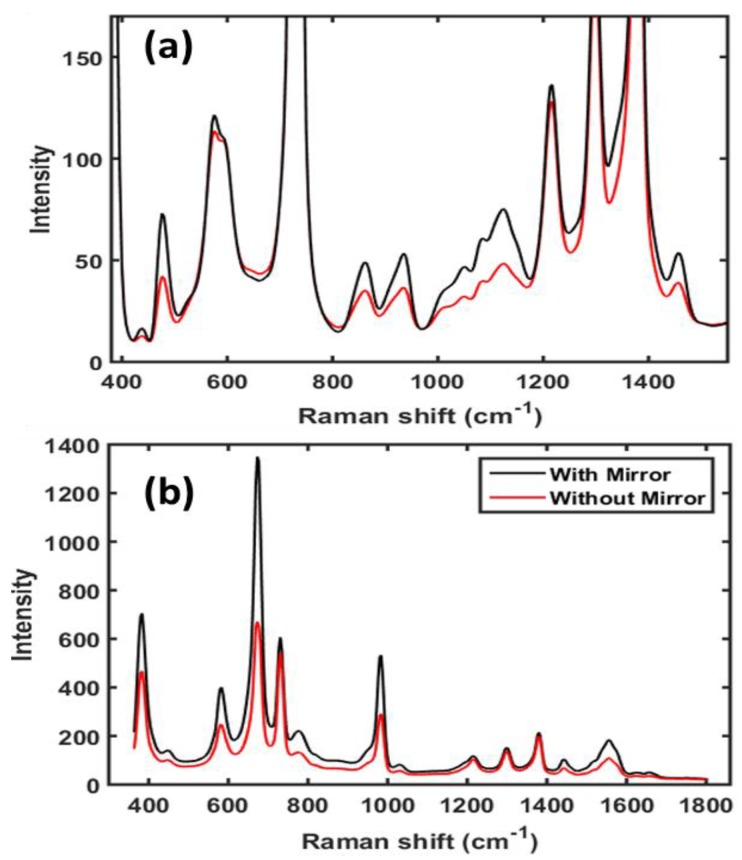
Preprocessed mean Raman spectra of central hundred pixels from S1 (**a**) and from S2 (**b**) collected with and without reflection mirror.

The Raman signal intensity for the subsurface layer of both S1 and S2 measured on the reflection mirror was considerably higher than that for samples measured without the mirror. This was expected, and due to the back-reflection of the Raman photons from the mirror’s surface as shown in Figure 1d. However, it is an interesting finding that there was no or only a very small difference in the Raman intensity of the superficial layer measured with or without the reflection mirror. This is also evident from the mean of Raman spectra of the central hundred pixels (Figure 5). Figure 5 shows that the spectra of the bottom layer collected on the reflection mirror are of high quality with a good S/N ratio. Visual inspection indicated a clear and obvious difference between the two spectra of subsurface materials collected with and without the mirror. The Raman bands associated with the subsurface material are comparatively high in intensity in both cases, however, no significant difference in band intensity was observed for surface material collected with and without the mirror. The results above were from replication 1, however, the results from the other replication were generally similar.

In practice, the mirror also reflects the excitation (Rayleigh) photons back through the sample where they are presented with a second opportunity for Raman scatter, thus generating more Raman photons to reach the detector and enhance the Raman signal. However, the signal enhancement due to double-pass will only be observable in transparent and thin samples where the laser focus is at the interface, and this increase would likely be lost in thick and opaque samples because the retro-reflected laser would not be effectively collected and collimated by the objective [12]. This effect is one of the possible explanations for the unnoticeable improvement in Raman intensity of the surface later. Another reason could be that the transmitted Raman photons from the surface layer experience losses due to the increased scattering and absorption by the subsurface layer and the double interaction with the subsurface layer: first while passing through the subsurface layer to the mirror, and second, when returning back from the mirror through the subsurface layer and to the detector. This is probably the case in transmittance Raman spectroscopy, where the Raman signals and fluorescence interference from the surface layer of the sample can be reduced because the excitation photons travel through the entire sample, and the transmitted Raman photons from the surface layer will interact with the next layer and (some of them) be absorbed.

**Figure 6:**
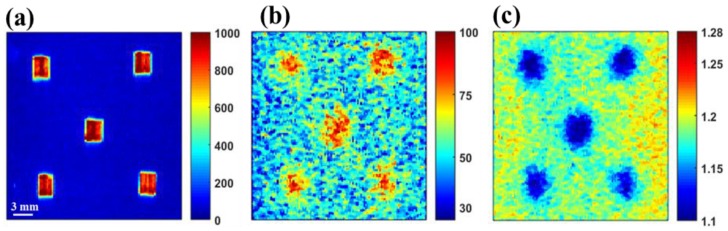
Raman band (1000 cm^−1^) images of polystyrene crystals exposed to the laser without any cover (**a**), covered with a 2 mm thick layer of wheat flour (**b**), and the spectral angle mapper (SAM) rule image of polystyrene crystals covered with a 2 mm thick layer of wheat flour (**c**). The intensity scales are in arbitrary units.

A second demonstration of the applicability of the reflection mirror to effectively recover the Raman signals of the small adulterant particles buried under the food powder was carried out. First, the penetration depth of the laser line in food powder (wheat flour) was determined by analyzing sample S3, which consisted of five polystyrene crystals (2 mm thick) covered by a 2 mm thick layer of wheat flour. The collected Raman image data was processed and the resulting images (Figure 6) reveal the presence of the hidden polystyrene crystals. Thus, the penetration depth of the laser line was confirmed as a minimum of 2 mm in wheat flour. The generated Raman band image (Figure 6b) and SAM rule image (Figure 6c) show the spatial location of the hidden polystyrene crystals, however, the spatial shape is slightly distorted when compared with Figure 6a, which is quite obvious because of the loss of spatial information with increasing depth, and also possibly due to diffusion of the detected Raman signals.

In the final example, sample S4 was analyzed. It contained tiny polystyrene pieces concealed by a 2 mm thick layer of wheat flour, which is a more likely scenario in the case of powder food adulteration (i.e., melamine in milk powder or bleaching agents in wheat flour). While the samples were arranged in a sample holder for Raman analysis, these adulterant particles can settle down to the bottom due to their relatively high density compared to food powder. Consequently, weak Raman signals from the adulterant particles at the bottom easily become swamped by the upper layer of food powder, and cannot be detected. Therefore, to demonstrate the effectiveness of the reflection mirror to reflect the transmitted Raman signals back from the adulterant particles and thus to form chemical images of the adulterant, the S4 specimen was prepared and mapped. Firstly, the effectiveness of the mirror application was evaluated by utilizing a single-band based univariate method for visualization of hidden polystyrene beads. However, the position of polystyrene beads was not visible in Raman band images of 1000 cm^−1^, thus, multivariate analysis tools were required to effective visualization of small polystyrene beads.

Since Raman imaging requires the effective extraction of chemical information from the corresponding data set, it can be achieved by a range of analytical methods. Raman imaging data for powdered food authenticity analysis are typically analyzed by two common methods: first, a blind source separation method such as principal component analysis (PCA) or independent component analysis, and second is the spectral similarity analysis method, such as spectral angle mapper (SAM) or spectral intensity divergence. However, these two different groups of data analysis methods exhibit both strengths and weaknesses, and thus testing using a single method can misinterpret the instrumental sensitivity. Therefore, Raman imaging data of specimen S4 was analyzed with two different methods, namely PCA and SAM. Both PCA and SAM are multivariate data analysis methods employing full spectra and are particularly useful when single band-based selective information is not available. The main difference lies in their nature, as PCA is an unsupervised method and SAM requires the assignment of a reference (adulterant) spectrum.

**Figure 7:**
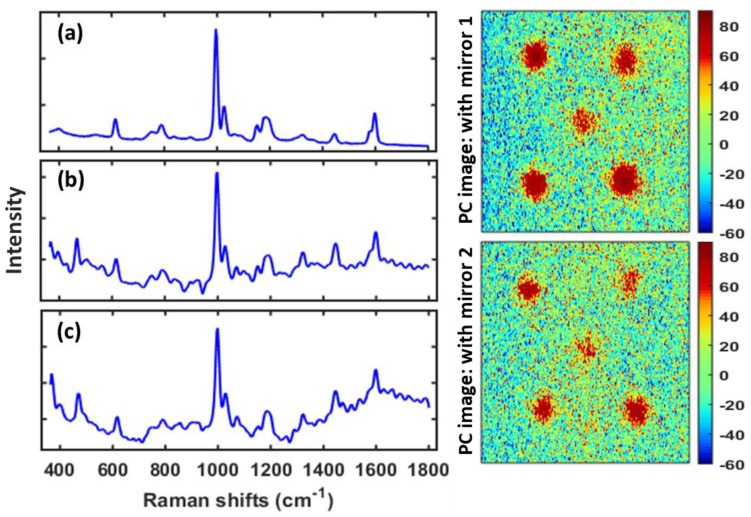
Mapping of hidden small polystyrene pieces using a reflection mirror: Raman spectra of polystyrene (**a**), and principle component analysis (PCA) loading plots calculated for S4 (with mirror) replications 1 (**b**) and 2 (**c**), and the corresponding PCA images on the right side. Note that the PCA results did not show any evidence of polystyrene pieces under wheat flour for the S4 data collected without the mirror.

**Figure 8:**
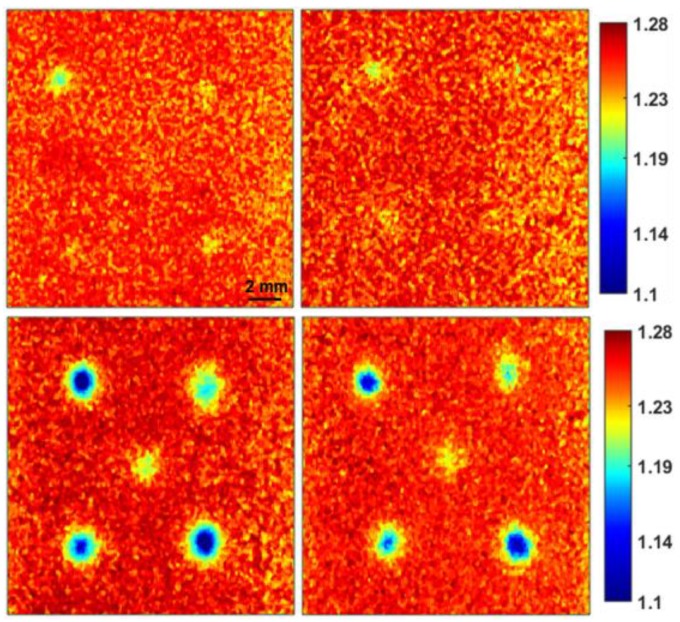
SAM generated Raman images of small polystyrene pieces mapped without (upper row) and with (lower row) the reflection mirror for two replicates. (Representation of Sample S4 with supervised analysis method SAM).

Figure 7 reports the results of the PCA analysis conducted on specimen S4 scanned with the reflection mirror. Figure 7a shows the Raman spectrum of polystyrene, and the calculated PCA loading plots are given in Figure 7b,c for replications 1 and 2, respectively. The Raman peaks of the concealed polystyrene are evident from the PCA loadings. The PCA images (distribution map) corresponding to the loadings were generated to illustrate the spatial position and reveal the concealed polystyrene pieces as shown in Figure 7. It should be noted that the concealed polystyrene pieces were not detected in specimen S4 when scanned without the reflection mirror. This is possibly because of the aforementioned reasons where the transmitted Raman signals from the hidden polystyrene pieces were reflected back by the background mirror toward the detector, thus contributing to the improvement in Raman intensity and S/N ratio, and eventually detected in the generated chemical images. In contrast, when the map was acquired with conventional Raman without the mirror, the relatively low signals which originated from the base materials were overwhelmed by the signals from the superficial layer and thus essentially lost. This is also apparent from the SAM generated rule images of the same specimen S4, where the concealed polystyrene pieces can be clearly visualized for data collected with the mirror (Figure 8). Nevertheless, unlike the PCA images of specimen S4 collected without the mirror, SAM rule images of the same sample show small evidence of hidden polystyrene (left upper part of the images on the upper row). This is probably because of the supervised nature of SAM analysis. Moreover, this particular piece of polystyrene was slightly thicker than the other pieces, therefore, it possibly generates a relatively higher amount of Raman signals, and thus it is detectable in the SAM rule images. It should be noted that the polystyrene piece buried at the center of the sample was the thinnest and relatively smaller in size, hence generating comparatively less Raman signals and is completely unnoticeable in conventional Raman images, but is evident when measured on the reflection mirror, though not as notably as the other hidden pieces of polystyrene.

Overall, the examples given here illustrate the potential of a reflection mirror underneath a sample to enhance the Raman signals of base materials. It should be noted that the reflection mirror underneath the sample actually works best as a reflector of transmitted Raman photons when the laser beam can penetrate through the whole depth of the sample and reach the reflection mirror. In the case where a laser cannot penetrate through the whole depth, no Raman signals of the base material can be reflected by the mirror. Hence, thin samples should be tested after confirming the penetration depth of the laser. In addition, the effect of the reflection mirror on Raman signals from different thin layers of a multiple layer specimen has yet to be tested.

## 4. Conclusions

In this report, the suitability and effectiveness of a reflection mirror is investigated for Raman imaging analysis of layered samples to determine whether or not the Raman signals of the subsurface layer can be enhanced. By using the reflection mirror underneath the sample, the results clearly show a significant improvement in signal from the subsurface layer, however, there was no notable enhancement in the Raman signal from the surface layer when the mirror was used. 

Additionally, by hiding the small pieces of polystyrene under wheat flour, it was demonstrated that adulterant particles which have settled at the very bottom of food powder can be effectively imaged using a reflection mirror, which is otherwise not possible with conventional backscattering Raman imaging. However, chemical adulterants or a subsurface layer with too small Raman scattering and a highly fluorescent surface layer can restrain notable enhancement in Raman signals when using a reflection mirror. In conclusion, the present work has several important implications for fast Raman imaging of a range of samples, as the total data collection time for Raman imaging can be reduced because of a high S/N ratio when using a reflection mirror, permitting more rapid mapping.
